# Michael Anthony Hession, FRCPsych

**DOI:** 10.1192/bjb.2025.10179

**Published:** 2026-04

**Authors:** Mary Hession

Formerly the founder of the *British Journal of Hospital Medicine* who competed in the London to Sydney Air Race in 1969.

**Figure:**
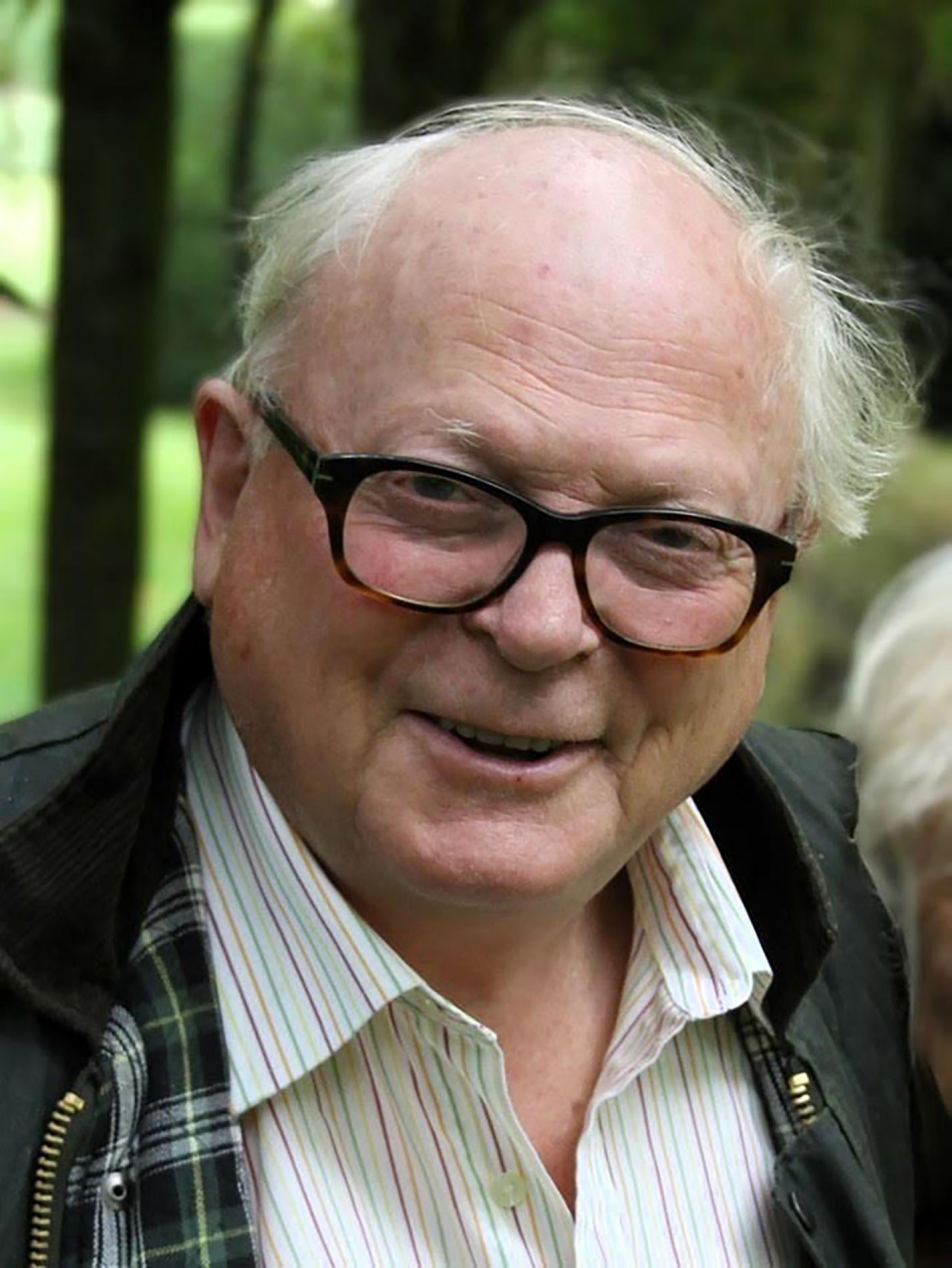

Michael Anthony Hession, who died on 27 December 2024, was born in Blackheath, south-east London, on 23 May 1939 to Roy and Phyllis (née Williams), both evangelical Christians. His parents sent him to the boarding school of St Lawrence College in Ramsgate, Kent, which had a strong evangelical Christian ethos. He described being beaten while a student there, claiming that it was a frequent and sadistic occurrence. Even worse, as his parents were regularly away preaching, he was often unable to return home during school holidays. These experiences in his formative years shaped his future self, including his choice of career.

Michael read medicine at Christ’s College Cambridge and then went to St Bartholomew’s Hospital, London. Here he met his wife Mary, who was studying law. They married when she was 20 and Michael 22.

After qualifying as a junior doctor in 1964, he found he was working more than 120 hours a week for £28 a month after deductions. This was unacceptable as it was risking junior doctors’ health as well as the health of their patients. He and a few others formed the Junior Hospital Doctors Action Committee and organised the first National Health Service doctors’ protest over pay and working conditions, lobbying the government, lodging a complaint with the British Medical Association (BMA) and getting their message out to a largely supportive press. He led the fight for representation for junior hospital doctors within the BMA.

In discussions with other hospital doctors, Michael realised there was a need for a magazine focusing on the interests of hospital doctors other than the existing academic medical journals. So, in 1966, he founded the *British Journal of Hospital Medicine*, a publication funded by advertisements that was distributed free to all doctors to enable them to keep up with new clinical research. The magazine quickly became successful, attracting offers to buy it. In 1969 it was sold to the Thomson Press. With the proceeds of the sale, Michael was able to learn to fly and then bought a twin-engine light aircraft and competed in the London to Sydney Air Race in December 1969.

He continued his medical training as a psychiatrist, at King’s College Hospital and the Maudsley Hospital, London. In 1974 he stood unsuccessfully as a Labour candidate in the Bromley Ravensbourne constituency. In his election leaflet Michael wrote that as a doctor the prescriptions he really wanted to write were for better housing, better education, freedom from poverty, and for a more sensitive and aware society. He continued his interest in politics, working closely with David Ennals (Secretary of State for Social Services 1976–1979), campaigning for changes to improve the rights of patients who had been detained under the Mental Health Act. Having suffered an unhappy childhood he undertook psychoanalysis to understand the effects it had on him and became a consultant child psychiatrist working for, among others, local authorities, the Church of England Children’s Society and the Institute of Child Psychology, Notting Hill, London.

In 1978 he moved to Herefordshire and worked as a general adult psychiatrist at the Mid Wales Hospital. He introduced psychotherapy and encouraged all members of staff to participate in a more therapeutic approach, where previously treatment was largely chemical with some physical interventions such as electroconvulsive therapy.

After he retired from hospital practice, he continued to sit on Mental Health Tribunals and worked as an independent expert in court proceedings.

Throughout his life Michael had a great love of music and played the cello and piano. Concerts were often held at the family home with celebrity musicians. Concert goers were also able to visit the garden where he had planted over 1000 trees. He is survived by his wife Mary, their children Lloyd, Rowena and Ruth, and seven grandchildren.

